# The Clustering of Low Diet Quality, Low Physical Fitness, and Unhealthy Sleep Pattern and Its Association with Changes in Cardiometabolic Risk Factors in Children

**DOI:** 10.3390/nu12020591

**Published:** 2020-02-24

**Authors:** Xianwen Shang, Yanping Li, Haiquan Xu, Qian Zhang, Ailing Liu, Guansheng Ma

**Affiliations:** 1 School of Behavioural and Health Sciences, Australian Catholic University, East Melbourne, VIC 3002, Australia; xianwen.shang@unimelb.edu.au; 2 Department of Medicine (Royal Melbourne Hospital), University of Melbourne, Parkville, VIC 3010, Australia; 3 Department of Nutrition, Harvard T.H. Chan School of Public Health, 655 Huntington Ave, Boston, MA 02115, USA; yanping@hsph.harvard.edu; 4 Institute of Food and Nutrition Development, Ministry of Agriculture and Rural Affairs, Beijing 100081, China; xuhaiquan@caas.cn; 5 National Institute for Nutrition and Health, Chinese Center for Disease Control and Prevention, Beijing 100050, China; zhangqian7208@163.com (Q.Z.); liuailing72@126.com (A.L.); 6 Department of Nutrition and Food Hygiene, School of Public Health, Peking University, Beijing 100191, China

**Keywords:** diet quality, sleep, cardiorespiratory fitness, clustering, cardiometabolic risk factors, children

## Abstract

The clustering of diet quality, physical activity, and sleep and its association with cardiometabolic risk (CMR) factors remains to be explored. We included 5315 children aged 6–13 years in the analysis. CMR score (CMRS) was computed by summing *Z*-scores of waist circumference, an average of systolic and diastolic blood pressure, fasting glucose, high-density lipoprotein cholesterol (multiplying by −1), and triglycerides. Low diet quality and low cardiorespiratory fitness (CRF) were more likely to be seen in a pair, but low diet quality was less likely to be clustered with unhealthy sleep patterns. Low diet quality, low CRF, and unhealthy sleep pattern was associated with a 0.63, 0.53, and 0.25 standard deviation (SD) higher increase in CMRS, respectively. Compared to children with no unhealthy factor (−0.79 SD), those with ≥1 unhealthy factor had a higher increase (−0.20 to 0.59 SD) in CMRS. A low diet quality-unhealthy sleep pattern resulted in the highest increase in CMRS, blood pressure, and triglycerides. A low diet quality–low CRF-unhealthy sleep pattern resulted in the highest increase in fatness and fasting glucose. Unhealthy factor cluster patterns are complex; however, their positive associations with changes in CMR factors are consistently significant in children. Some specific patterns are more harmful than others for cardiometabolic health.

## 1. Introduction

Unhealthy diet quality, low levels of physical activity, and unhealthy sleep patterns are commonly seen in school children [1,2]. Some study has shown that physical activity in leisure-time was associated with healthier eating habits [3]; however, more other studies did not find significant associations between physical activity and diet habits [2,4,5]. Evidence from cross-sectional studies has demonstrated that unhealthy sleep pattern, defined by short sleep duration and later bedtimes, is associated with poor diet quality [6] and physical inactivity [7]. However, data from longitudinal studies on the effect of sleep patterns on diet and physical activity are limited [5,6]. Although previous studies have investigated the clustering of multiple health behaviors, the patterns are mixed, and more studies are needed to identify major patterns among children [1,2].

Increasing evidence has highlighted the importance of diet, physical activity, and sleep on metabolic health in children [1,2,8,9]. Studies have shown that Western dietary patterns, high energy-dense patterns, or sweet dietary patterns were associated with a higher risk of cardiometabolic risk (CMR) factors [10,11,12], whereas vegetable and wholemeal patterns were associated with favorable changes in CMR factors [13]. Cardiorespiratory fitness (CRF) represents physical activity habits [14] and is an indicator of accumulated physical activity in the long-term [15]. Given CRF can be measured accurately, it is a stronger predictor of CMR factors than physical activity in both children and adults [16,17,18]. A recent systematic review reported that shorter sleep duration was associated with higher CMR in children [9], whilst other studies also suggest late bedtimes were associated with adverse metabolic risk outcomes [6,8]. Although these health factors have been individually linked to metabolic health, the association between the clustering of multiple health factors and CMR factors is less known [1,2,7]. Determining the clustering of multiple health factors may help target the factors that are best selected for promoting health [1].

The present study examined which two of low diet quality, low CRF, and unhealthy sleep patterns are more likely to be clustered in pairs. We also examined whether some combinations of these unhealthy factors were associated with a higher increase in CMR factors in children.

## 2. Materials and Methods

### 2.1. Participants

This analysis was based on the Nutrition-based Comprehensive Intervention Study On Childhood Obesity in China, and the study has been detailed elsewhere [19]. Briefly, the study was conducted in six capital or province capital cities including Beijing, Shanghai, Chongqing, Jinan, Harbin, and Guangzhou. A total of 9901 children from 390 classes within 38 schools were screened for eligibility. Among 9867 children who were assessed at baseline (May 2009), 8572 were reassessed at follow-up (May 2010). In addition, 5315 children with diet, CRF, sleep, and CMR markers assessed were included in the final analysis (Supplementary Figure S1).

The study protocol was approved by the Ethical Review Committee of the National Institute for Nutrition and Food Safety, Chinese Centre for Disease Control and Prevention. Oral assent was collected from children whilst written informed consent was obtained from the next of kin, carers, or guardians of all participants.

### 2.2. Dietary Assessment

Dietary intake was assessed using 24-h diet recalls for three consecutive days (two weekdays and one weekend day) in children of grades 2–5. During the interview, samples of local household dishes and utensils (different sizes of bowls, plates, and spoons) were displayed to the children. They were then shown pictures of common foods eaten in these dishes or utensils to indicate portion size consumed. Diet intake was recalled immediately after each meal to ensure the accuracy of the assessment. 

The trained interviewer and the tutor would help children recall food intake at school while parents would help recall foods consumed at home. Nutrients and energy intake was calculated based on the China Food Composition [20]. Energy adjusted food consumption per day was computed as ([100×weight in grams]/total energy intake in Kcal). We used a validated healthy dietary score in our previous work, which was calculated based on nine food groups: refined grains (<median), fish (>median), fried foods (<median), sugar-sweetened beverages (<median), rice (>median), wheat (<median), fungi and algae (<median), roots and tubers (<median), and red meat other than pork (>median). The total score ranged between 0–9 and we defined low diet quality as score ≤5 for boys and ≤3 for girls. This cut-off point was derived using the general linear regression model (GLM) for boys and girls separately. We examined the association between diet quality and change in CMR score (CMRS) with restricted cubic splines. Knots were then derived, and individuals were divided into groups accordingly. We used the knot that yielded the largest difference in CMRS change between two groups with each group accounting for at least 5% of the total population as the cut-off point for diet quality.

### 2.3. Physical Fitness Measurements

The 50-m shuttles run, as a traditional test in China, was used to evaluate CRF among children [21]. A physical education teacher and researcher were involved in the test. Before the test, they would check if the children were under good health conditions. This test required children to run back and forth four times at their highest speed along a track between two poles set 50-m apart and to turn around the poles counterclockwise. The test was recorded to the nearest 0.1 s (CASIO, HS-70W stopwatch). The results would be accounted for as a component of the performance assessment of physical education for the semester to motivate children to try their best in the test. We used the speed (400 m divided by test time in seconds) as CRF with a higher value indicating better performance. Age- and sex-standardized Z score of CRF was calculated as (value−mean)/standard deviation (SD). Low CRF was defined as CRF Z score≤0 (the cut-off point was derived using the same method as that for diet quality) for both girls and boys.

### 2.4. Sleep Pattern

Sleep pattern was assessed using a validated 7-day Physical Activity Recall questionnaire [22]. Children were asked about sleep duration via four items: “On school days, what time do you usually go to sleep the night in last week?”; “What time do you usually wake up in the morning last week?”; “On weekends, what time do you usually go to sleep the night in last week?”; and “What time do you usually wake up in the morning last week?”. Hours of sleep per night on weekdays and weekends were computed as the sum of total hours of sleep on weekdays and those on weekends divided by 7. Unhealthy sleep patterns were defined by sleep duration less than 9 h or greater than 12 h or late bedtimes (after 9:00 p.m.) [23,24].

### 2.5. Confounders

Puberty status was recorded by investigators during the interview when physical examinations were conducted. Physical activity was assessed using a validated physical activity questionnaire, from which metabolic equivalent was calculated [22]. Birthweight, household income, parental education, and parental height and weight were reported by parents using a self-administered questionnaire.

### 2.6. Physical Examinations and Blood Tests

Physical examinations and blood tests (10–14 h fasting beforehand) were performed at both baseline and follow-up following standardized procedures by trained staff.

Height was measured to the nearest 0.1 cm using a freestanding stadiometer and weight was measured to the nearest 0.1 kg using a balance-beam scale. Body mass index (BMI) was computed as weight in kilograms divided by the square of height in meters. Waist circumference (WC) was measured midway between the lowest rib and the superior border of the iliac crest on expiration to the nearest 0.1 cm, and the average of two measurements was used.

Systolic and diastolic blood pressure (SBP and DBP) was measured in the seated position using a mercury sphygmomanometer (XJ300/40-1, Shanghai, China) by trained nurses with at least 10 min rest before the measurement. Three measurements were taken to the nearest two mmHg and the average of the last two measurements was used. Mean arterial pressure (MAP) was calculated as (DBP + 0.33 × [SBP−DBP]).

A single frequency (50 Hz) hand to foot bioelectrical impendence device (ImpDF50, Impedimed Pty Ltd., Qld, City, Australia), with subjects in a calm state, was used to assess body composition. Body fat mass was computed using the prediction formula developed by Deurenberg et al. [25], and percent body fat (PBF) was calculated as fat mass divided by body weight.

Fasting blood samples (5 mL) were collected in serum separator tubes in the morning after 10–14 h of overnight fast and then transported, lifted to be clotted for 20–30 min and centrifuged for 10–15 min at 3200 rpm using a Multifuge 35R. Blood samples for glucose tests were transported to the clinical laboratory in a cooler with an ice block at 4 °C. Other blood samples were stored in a deep freezer at −70 °C until further testing. Fasting glucose was measured using the glucose-oxidize method (Daiichi Pharmaceutical Co., Ltd, Tokyo, Japan) within three hours after the fasting blood sample was obtained. Fasting insulin was measured using the immunoenzymatic method (analyzer AXSYM, Abbott Co., Ltd, City, Japan).

Conventional enzymatic assays were used to measure levels of serum triglycerides (TG), total cholesterol (TC), high-density lipoprotein cholesterol (HDL-C), and low-density lipoprotein cholesterol (LDL-C) with a 7080 Automatic Analyzer (Daiichi Pharmaceutical Co., Ltd, Tokyo, Japan).

### 2.7. Statistical Analysis

BMI, WC, PBF, SBP, DBP, MAP, TC, HDL-C, LDL-C, TG, fasting glucose, and insulin at baseline and follow-up were standardized (i.e., *Z* scores were calculated: *Z* = (value−mean)/SD using sex and age-specific means and SDs). CMRS was calculated by summing *Z* scores of WC, the average of SBP and DBP, fasting glucose, HDL-C (multiplying by −1), and TG [26] with a higher score representing lower metabolic health. 

We used the logistic regression model to test if two of the three unhealthy factors were more likely to be clustered in pairs. Age, sex, intervention, puberty, birth weight, BMI, and energy intake at baseline, household income, parental BMI, and parental education were adjusted for in the multivariable analysis.

According to the combination of the three health factors, children were divided into eight groups as those with no unhealthy factor, low diet quality only, low CRF only, unhealthy sleep pattern only, low diet quality-low CRF, low diet quality-unhealthy sleep pattern, low CRF-unhealthy sleep pattern, and all three unhealthy factors. ANOVA for continuous variables and chi-square tests for categorical variables were performed to compare the difference of baseline characteristics across the combination of three unhealthy factors.

Given that the interaction between sex/intervention and a combination of unhealthy factors with changes in most CMR factors was not significant (Supplementary Tables S1 and S2), we conducted the main analysis in the whole population. GLM was used to test the difference of changes in CMR factors between individuals with a different combination of three healthy factors. We tested the following models: (1) age and sex (geographic factors) as fixed effect and clustering effect of children within classes in schools as random effect; (2) model 1 plus puberty, grade, intervention, corresponding CMR factor, BMI, physical activity, and intake of energy, vegetable, fruit, pork, nuts and legumes at baseline (physiological and behavioral factors); (3) model 2 plus birth weight, breastfeeding, household income, or parental BMI and education (socioeconomic and predisposing factors). We used the Benjamin–Hochberg procedure to control the false discovery rate at level 5% for multiple comparisons [27]. Bonferroni *p*-value adjustments were performed for all pairwise comparisons. We also analyzed the association of change in the number of unhealthy factors with changes in CMR factors using GLM. Missing values for categorical confounders were assigned as a single category whilst means were given to missing values for continuous confounders.

Sensitivity analysis was performed to test the association between combinations of unhealthy factors and changes in CMR factors in the control group.

Data analyses were conducted using SAS 9.4 for Windows (SAS Institute Inc., City, US State abbrev., USA) and all *p*-values were two-sided.

## 3. Results

### 3.1. Characteristics of Participants

In the final analysis, 5315 children (48.7% boys) aged 6–13 years (mean ± SD: 9.55 ± 1.19) were included. Compared with girls, boys were more likely to have low diet quality and two or three unhealthy factors. Children with three unhealthy factors were more likely to be older, have higher BMI, WC, SBP, DBP, and MAP but lower LDL-C than those with no unhealthy factor (all *p*-values < 0.05, Table 1). Children with high birth weight were more likely to have three unhealthy factors than those with low birth weight. Children whose mother or father with low BMI were less likely to have three unhealthy factors, whereas those whose mother or father had a low amount of education were more likely to have three unhealthy factors (Supplementary Table S3).

### 3.2. The Clustering of Unhealthy Factors

The prevalence of low diet quality, low CRF, and unhealthy sleep patterns was 34.8%, 59.6%, and 46.4%, respectively. Children with low CRF (41.4%) were more likely (odds ratio (OR) (95% confidence interval (CI)): 2.43 (2.11–2.79), *p*-value < 0.0001) to have low diet quality than those with high CRF (25.1%) in the cross-sectional analysis. Children with unhealthy sleep patterns at baseline (32.8%) were less likely to have low diet quality (OR (95% CI): 0.79 (0.69–0.90)) compared to those with healthy sleep patterns (36.5%). Low CRF at baseline was associated with a higher likelihood of low diet quality (OR (95% CI): 1.60 (1.33–1.91)), but a lower likelihood of unhealthy sleep pattern (0.75 (0.66–0.84)) at follow-up. An unhealthy sleep pattern at baseline was associated with a lower likelihood of low diet quality (OR (95% CI): 0.80 (0.67–0.95)), but a higher likelihood of low CRF (1.52 (1.30–1.77)) at follow-up. Children with low diet quality at baseline were less likely to have unhealthy sleep patterns at follow-up (OR (95% CI): 0.57 (0.50–0.66)) compared to those with high diet quality (Table 2).

### 3.3. Individual Unhealthy Factors and Changes in CMRS

The multivariable-adjusted CMRS increased 0.15 ± 0.14 SD over one year in children. Low diet quality was positively associated with the change in CMRS after adjustment for confounders and the adjusted difference in CMRS change between individuals with low and high diet quality was 0.63 SD (0.43 ± 0.14 SD for low diet quality, −0.20 ± 0.14 SD for high diet quality). Low CRF resulted in a 0.53 SD higher increase in CMRS (0.23 ± 0.13 SD for low CRF, −0.30 ± 0.14 SD for high CRF). Children with unhealthy sleep patterns had a 0.25 SD higher increase in CMRS than those with healthy sleep patterns (0.20 ± 0.14 SD for unhealthy sleep pattern, −0.05 ± 0.14 SD for healthy sleep pattern) (Table 3).

### 3.4. Clustering of Unhealthy Factors and Changes in CMRS

Children with no unhealthy factor had the highest decrease in CMRS (−0.79 ± 0.15 SD), compared to those with one or more unhealthy factors. Low diet quality-unhealthy sleep pattern resulted in the highest increase in CMRS (0.59 ± 0.19 SD), followed by low diet quality-low CRF (0.41 ± 0.15 SD), low diet quality-low CRF-unhealthy sleep pattern (0.38 ± 0.16 SD), and low CRF-unhealthy sleep pattern (0.07 ± 0.15 SD). Low diet quality-low CRF-unhealthy sleep pattern resulted in the highest increase in BMI, WC, PBF, and fasting glucose and the lowest increase in HDL-C. Low diet quality-unhealthy sleep patterns resulted in the highest increase in SBP, DBP, MAP, and TG. Children with unhealthy sleep patterns only had the highest increase in TC, LDL-C, and insulin (Table 4). These results were consistent with those for Model 1 and Model 2 (Supplementary Table S4).

A decrease in the number of unhealthy factors was associated with favorable changes in CMRS, BMI, WC, TG, and HDL-C after adjustment for confounders (Supplementary Table S5).

### 3.5. Sensitivity Analysis

Similar results for the association between clustering of unhealthy factors and changes in CMR factors were observed in children in the control group compared with those in the whole population (Supplementary Table S6).

## 4. Discussion

We found children with low CRF at baseline were more likely to have low diet quality at both baseline and follow-up, but low diet quality was less likely to be clustered with unhealthy sleep patterns. There was a bidirectional association between CRF and sleep patterns. Compared with children with no unhealthy factors, those with one or more unhealthy factors had a higher increase in CMRS. Low diet quality-unhealthy sleep patterns resulted in the highest increase in CMRS, SBP, DBP, MAP, and TG. Low diet quality-low CRF-unhealthy sleep pattern resulted in the highest increase in BMI, WC, PBF, and fasting glucose and the lowest increase in HDL-C.

Our findings agree with previous studies showing that unhealthy factors are prevalent among children [1,2]. We found that low diet quality was positively associated with low CRF. Likely, a cross-sectional analysis of 279 New Zealand adolescents aged 14–18 years reported that CRF is positively associated with a healthy dietary pattern [28]. However, no significant associations between dietary patterns and CRF were found in another cross-sectional study of 398 New Zealand children aged 9–11 years [29]. Our further analysis showed that children with low baseline CRF were more likely to have low diet quality at follow-up compared to those with high baseline CRF, whereas a baseline healthy diet score was not significantly associated with CRF at follow-up. Given that this association has not been reported in longitudinal studies, future research needs to examine this association. Two cross-sectional studies have shown that unhealthy sleep patterns were associated with low diet quality in children [30,31]. This is inconsistent with our findings demonstrating that unhealthy sleep patterns and low diet quality were less likely to be seen in a pair. The inconsistent findings may be attributed to different food items used in the assessment of diet quality. Although sugar-sweetened beverages, fried foods, fish, and whole grains were included in the assessment of dietary score in all studies, a healthy diet score was calculated with refined grains as one component in our study given its strong association with CMRS. In addition, we did not involve vegetable and fruit intake in our scoring since they were not among the leading predictors of CMRS. Our longitudinal analysis showed that unhealthy sleep pattern at baseline was associated with a higher likelihood of low diet quality at follow-up and vice versa. Our findings suggest unhealthy sleep patterns and low diet quality were less likely to be seen in a pair and more longitudinal studies need to explore whether some nutrients or foods were strongly correlated to sleep patterns. There has been inconsistent information regarding the association between physical activity and sleep patterns in children [32]. We found children with unhealthy sleep patterns were more likely to have low CRF at follow-up, but low CRF at baseline was inversely associated with unhealthy sleep patterns at follow-up. This is supported by the data from 5779 children aged 9–11 years, showing that an increase in sleep duration resulted in an increased moderate and vigorous physical activity the following day, whereas an increase in moderate and vigorous physical activity was associated with a decreased sleep duration the following day [33]. However, other studies did not find this bidirectional association [34,35]. Our study also demonstrated that children being male or older, or having high birth weight, high parental BMI, and low parental education were more likely to have three unhealthy factors suggesting these socioeconomic or family factors may be key mediators for the clustering of unhealthy factors.

Previous studies have focused on diet and physical activity intervention for the prevention of obesity and related CMR [36]; however, recent evidence suggests sleep may be included as part of the lifestyle package for intervention [6]. Our findings suggest that children with any one or more unhealthy factors at baseline had a significantly higher increase in CMRS compared with those who were free of unhealthy factors. When analyzed individually, unhealthy sleep patterns resulted in a significant increase in CMRS, even though the magnitude is smaller than low diet quality or low CRF alone. Although unhealthy sleep patterns and low diet quality were less likely to be clustered in a pair, children with these two unhealthy factors had the highest increase in CMRS, blood pressure and TG compared to those with other combinations of unhealthy factors. This highlights the importance of targeting unhealthy sleep patterns and low diet quality as intervention priorities for the prevention of obesity-related CMR. Furthermore, unhealthy sleep patterns only among eight combinations of three unhealthy factors resulted in the highest increase in TC, LDL-C, and insulin indicating sleep is an important factor for lipids and insulin metabolism in children.

We also found that low diet quality-low CRF was the second most harmful combination after low diet quality-unhealthy sleep patterns for cardiometabolic health. This is in line with previous studies showing that combined diet and physical activity intervention yielded a significant beneficial effect on the prevention of obesity in children [36]. A cross-sectional study of 415 Spanish children and adolescents showed that a low physical activity-poor diet lifestyle pattern was not significantly associated with overweight (prevalence ratio: 2.00 (95% CI: 0.87–4.86) compared with the healthy lifestyle pattern [37], which might be due to the small sample size of their study. Whether clustering of low diet quality and low CRF was associated with higher CMR needs to be warranted in more longitudinal studies. Low diet quality-low CRF-unhealthy sleep pattern should not be overlooked since this combination resulted in the highest increase in BMI, WC, PBF, fasting glucose, and the lowest increase in HDL-C in our study. Evidence on whether to increase healthy behaviors or decrease unhealthy behaviors for promoting health is inconclusive [1]. Our study showed that one unhealthy factor reduction was associated with a 0.54 SD decrease in CMRS among children suggesting that to reduce unhealthy behaviors may be more efficient for CMR reduction. Although unhealthy factor cluster patterns are complex [2], their positive associations with CMR factors are consistently significant and low diet quality-unhealthy sleep patterns, low diet quality-low CRF, and low diet quality-low CRF-unhealthy sleep pattern resulted in the highest increase in CMRS in children.

The strengths of the present study included the large sample size and the assessment of unhealthy factors and multiple CMR factors at both baseline and follow-up. To our knowledge, this is the first study to investigate the association between clustering of low diet quality, low CRF, and unhealthy sleep pattern and changes in multiple CMR factors in a longitudinal analysis. There are also several limitations to the present study. The follow-up period is relatively short to judge the effect of unhealthy factors on change in CMR and longitudinal studies with long-term follow-up are needed to warrant our findings. Furthermore, because of the observational nature of the analysis in the present study, causal relations could not be established based on our findings. Thirdly, we used CRF to represent levels of physical activity because the accelerator was used to assess physical activity in a subgroup only of our study. However, CRF is a more reliable indicator of a long-term status of exercise [14,15] compared with objectively measured physical activity in seven days only. Fourthly, there may be potential influences of self-reported sleep patterns and diet quality in our study; however, the measurement errors would be more likely to bias true associations to the null as the data were collected before the CMR markers were measured at follow-up. Fifthly, the study was conducted in children in urban areas such that the findings might not be generalized to those in rural areas.

## 5. Conclusions

Unhealthy factor cluster patterns are complex; however, their positive associations with changes in CMR factors are consistently significant in children. Low diet quality-unhealthy sleep pattern, low diet quality-low CRF, and low diet quality-low CRF-unhealthy sleep pattern are the most harmful combinations for cardiometabolic health. Intervention studies targeting these combinations are needed to warrant our findings.

## Figures and Tables

**Table 1:** Baseline characteristics by a combination of three unhealthy factors.

	No Unhealthy Factor	Low Diet Quality Only	Low CRF Only	Unhealthy Sleep Pattern Only	Low CRF-Unhealthy Sleep Pattern	Low Diet Quality-Unhealthy Sleep Pattern	Low Diet Quality-Low CRF	Three Unhealthy Factors	*p*-Value *
Age (years)	9.24 ± 1.02 ^†^	9.35 ± 1.16	9.27 ± 1.11	9.77 ± 1.19	9.76 ± 1.20	9.83 ± 1.25	9.57 ± 1.22	9.79 ± 1.25	<0.0001
Sex									<0.0001
Boys	311 (36.1) ^‡^	263 (84.0)	290 (30.6)	312 (41.3)	278 (30.9)	183 (79.6)	534 (73.4)	418 (72.3)	
Girls	550 (63.9)	50 (16.0)	658 (69.4)	444 (58.7)	623 (69.1)	47 (20.4)	194 (26.6)	160 (27.7)	
Puberty									0.95
No	818 (95.0)	302 (96.5)	872 (92.0)	646 (85.4)	796 (88.3)	212 (92.2)	690 (94.8)	550 (95.2)	
Yes	43 (5.0)	11 (3.5)	76 (8.0)	110 (14.6)	105 (11.7)	18 (7.8)	38 (5.2)	28 (4.8)	
Body composition									
BMI (kg/m^2^)	16.40 ± 2.52	16.98 ± 2.54	17.34 ± 3.29	16.41 ± 2.37	17.51 ± 3.32	17.10 ± 3.06	17.70 ± 3.49	18.19 ± 3.82	<0.0001
WC (cm)	55.58 ± 6.70	57.74 ± 7.15	58.39 ± 9.10	56.54 ± 6.56	59.76 ± 9.29	58.55 ± 8.39	59.95 ± 9.71	61.83 ± 10.83	<0.0001
PBF (%)	23.09 ± 4.59	22.26 ± 4.31	25.34 ± 4.63	22.35 ± 4.54	25.17 ± 4.80	21.86 ± 4.84	24.24 ± 4.78	24.46 ± 5.02	<0.0001
Blood pressure									
SBP (mm Hg)	99.37 ± 10.35	99.11 ± 10.96	100.38 ± 10.78	99.76 ± 10.84	101.23 ± 10.69	100.29 ± 11.46	101.73 ± 10.80	102.23 ± 10.46	<0.0001
DBP (mm Hg)	61.98 ± 7.78	61.75 ± 8.48	63.89 ± 8.90	63.96 ± 9.49	65.99 ± 9.46	62.86 ± 9.45	65.07 ± 9.14	65.36 ± 8.66	<0.0001
MAP (mm Hg)	74.43 ± 7.98	74.19 ± 8.60	76.04 ± 8.73	75.88 ± 9.09	77.73 ± 9.07	75.32 ± 9.31	77.26 ± 9.01	77.64 ± 8.58	<0.0001
Lipids									
TC (mmol/L)	3.96 ± 0.80	3.92 ± 0.81	4.05 ± 0.73	4.30 ± 0.86	4.30 ± 0.85	4.04 ± 0.79	3.97 ± 0.68	3.94 ± 0.68	0.68
HDL-C (mmol/L)	1.45 ± 0.29	1.48 ± 0.32	1.44 ± 0.29	1.51 ± 0.31	1.49 ± 0.32	1.49 ± 0.28	1.45 ± 0.30	1.44 ± 0.31	0.78
LDL-C (mmol/L)	2.23 ± 0.60	2.20 ± 0.62	2.16 ± 0.65	2.23 ± 0.63	2.19 ± 0.65	2.14 ± 0.63	1.94 ± 0.63	1.92 ± 0.60	<0.0001
TG (mmol/L)	0.84 ± 0.46	0.83 ± 0.48	0.83 ± 0.42	0.81 ± 0.40	0.85 ± 0.47	0.72 ± 0.34	0.80 ± 0.42	0.83 ± 0.51	0.0927
Glucose and insulin									
Fasting glucose (mmol/L)	4.58 ± 0.52	4.62 ± 0.60	4.47 ± 0.56	4.61 ± 0.45	4.51 ± 0.55	4.62 ± 0.53	4.44 ± 0.57	4.52 ± 0.59	0.0001
Log insulin	1.57 ± 0.55	1.51 ± 0.53	1.62 ± 0.64	1.67 ± 0.60	1.81 ± 0.65	1.62 ± 0.56	1.61 ± 0.60	1.65 ± 0.63	0.0004
CMRS	−0.17 ± 2.27	−0.29 ± 2.57	−0.08 ± 2.41	−0.46 ± 2.31	−0.14 ± 2.37	−0.51 ± 2.18	−0.20 ± 2.40	0.07 ± 2.57	0.35
Physical activity (MET/week)	643.3 ± 597.6	753.2 ± 681.0	652.4 ± 596.2	557.9 ± 590.8	606.7 ± 598.4	639.5 ± 631.8	655.9 ± 491.6	609.5 ± 488.2	0.14
Dietary intake									
Energy (kcal/day)	1176.78 ± 530.43	1377.66 ± 543.52	1193.11 ± 569.28	1385.63 ± 589.20	1305.41 ± 613.17	1484.70 ± 644.60	1222.03 ± 578.79	1258.06 ± 562.18	0.0164
Protein intake (g/100 Kcal/day)	4.69 ± 1.24	4.25 ± 1.05	4.36 ± 1.25	4.51 ± 1.18	4.29 ± 1.08	4.13 ± 1.01	3.99 ± 0.87	4.13 ± 0.93	<0.0001
Fat intake (g/100 Kcal/day)	2.93 ± 1.08	2.87 ± 1.10	2.95 ± 1.25	2.88 ± 1.06	2.90 ± 1.13	2.83 ± 1.15	3.06 ± 1.20	3.10 ± 1.19	0.0027
Carbohydrate intake (g/100 Kcal/day)	13.82 ± 2.93	14.40 ± 2.77	14.15 ± 3.20	14.15 ± 2.82	14.37 ± 2.91	14.64 ± 2.77	14.30 ± 2.90	14.10 ± 2.92	0.0105
Fibre intake (g/100 Kcal/day)	0.51 ± 0.29	0.57 ± 0.32	0.52 ± 0.34	0.47 ± 0.27	0.48 ± 0.26	0.57 ± 0.31	0.59 ± 0.44	0.59 ± 0.36	<0.0001

BMI, body mass index; CMRS, cardiometabolic risk score; DBP, diastolic blood pressure; HDL-C, high-density lipoprotein cholesterol; LDL-C, low-density lipoprotein cholesterol; MAP, mean arterial pressure; MET, metabolic equivalent; PBF, percent body fat; SBP, systolic blood pressure; TC, total cholesterol; TG, triglyceride; WC, waist circumference. *ANOVA was used to test the difference of continuous variables across a combination of three unhealthy factors and chi-square for categorical variables. †All such data were mean ± standard deviation. ‡All such data were frequency (percentage). Birth weight, parental BMI, parental education, household income, and intervention by a combination of three unhealthy factors were shown in Supplementary Table S3.

**Table 2:** Associations between unhealthy factors.

	Healthy Factor (Participants)	Unhealthy Factor (Participants)	Percentage of Unhealthy Factor	Multivariable-Adjusted Analysis ^*^	
				OR (95% CI)	*p*-Value
CRF at baseline	High diet quality at baseline	Low diet quality at baseline			
High	1617	543	25.14	Reference	<0.0001
Low	1849	1306	41.39	2.43 (2.11–2.79)	
CRF at baseline	High diet quality at follow-up	Low diet quality at follow-up			
High	1452	305	17.36	Reference	<0.0001
Low	1691	552	24.61	1.60 (1.33–1.91)	
CRF at baseline	Healthy sleep pattern at baseline	Unhealthy sleep pattern at baseline			
High	1174	986	45.65	Reference	0.12
Low	1676	1479	46.88	1.10 (0.98–1.23)	
CRF at baseline	Healthy sleep pattern at follow-up	Unhealthy sleep pattern at follow-up			
High	1035	1000	49.14	Reference	<0.0001
Low	1667	1182	41.49	0.75 (0.66–0.84)	
CRF at baseline	High CRF at follow-up	Low CRF at follow-up			
High	1243	497	28.56	Reference	<0.0001
Low	551	1011	64.72	4.24 (3.62–4.96)	
Sleep pattern at baseline	High diet quality at baseline	Low diet quality at baseline			
Healthy	1809	1041	36.53	Reference	0.00042
Unhealthy	1657	808	32.78	0.79 (0.69–0.90)	
Sleep pattern at baseline	High diet quality at follow-up	Low diet quality at follow-up			
Healthy	1670	492	22.76	Reference	0.01178
Unhealthy	1473	365	19.86	0.80 (0.67–0.95)	
Sleep pattern at baseline	High CRF at follow-up	Low CRF at follow-up			
Healthy	1013	722	41.61	Reference	<0.0001
Unhealthy	781	786	50.16	1.52 (1.30–1.77)	
Sleep pattern at baseline	Healthy sleep pattern at follow-up	Unhealthy sleep pattern at follow-up			
Healthy	1921	695	26.57	Reference	<0.0001
Unhealthy	781	1487	65.56	4.78 (4.21–5.44)	
Diet quality at baseline	High CRF at follow-up	Low CRF at follow-up			
High	1326	1110	45.57	Reference	0.23
Low	468	398	45.96	0.89 (0.75–1.07)	
Diet quality at baseline	Healthy sleep pattern at follow-up	Unhealthy sleep pattern at follow-up			
High	1681	1551	47.99	Reference	<0.0001
Low	1021	631	38.20	0.57 (0.50–0.66)	
Diet quality at baseline	High diet quality at follow-up	Low diet quality at follow-up			
High	2415	358	12.91	Reference	<0.0001
Low	728	499	40.67	2.65 (2.22–3.17)	

CRF, cardiorespiratory fitness; OR, odds ratio; CI, confidence interval. * Logistic regression was used to estimate Odds Ratios and 95% confidence intervals. Multivariable-model was adjusted for age, sex, intervention, puberty, birth weight, BMI, energy intake at baseline, household income, parental BMI, and parental education.

**Table 3:** Diet quality, cardiorespiratory fitness, and sleep pattern and changes in cardiometabolic risk score in children.

	Change in CMRS		*p*-Value *
	Healthy	Unhealthy	
Diet quality			
Participants	2979	1533	
Mean ± SE, Model 1 ^†^	−0.43 ± 0.11	0.31 ± 0.12	<0.0001
Mean ± SE, Model 2 ^‡^	−0.29 ± 0.11	0.35 ± 0.11	<0.0001
Mean ± SE, Model 3 ^§^	−0.20 ± 0.14	0.43 ± 0.14	<0.0001
Cardiorespiratory fitness			
Participants	1891	2621	
Mean ± SE, Model 1	−0.62 ± 0.12	0.12 ± 0.11	<0.0001
Mean ± SE, Model 2	−0.37 ± 0.11	0.15 ± 0.10	<0.0001
Mean ± SE, Model 3	−0.30 ± 0.14	0.23 ± 0.13	<0.0001
Sleep pattern			
Participants	2425	2087	
Mean ± SE, Model 1	−0.28 ± 0.12	-0.01 ± 0.12	0.0001
Mean ± SE, Model 2	−0.16 ± 0.11	0.08 ± 0.11	0.0002
Mean ± SE, Model 3	−0.05 ± 0.14	0.20 ± 0.14	0.0001

* General linear regression model (GLM) was used to examine the association of individual unhealthy factors with changes in CMR factors. ^†^ Model 1 was adjusted for age, sex, corresponding CMR factor at baseline as fixed effects and clustering effect of children within classes in schools as random effects. ^‡^ Model 2 was adjusted for model 1 plus intervention group, puberty, grade, BMI, physical activity, and intake of energy, vegetable, fruit, pork, nuts and legumes at baseline. ^§^ Model 3 was adjusted for model 2 plus birth weight, breastfeeding, household income, or parental BMI and education.

**Table 4:** Combination of three healthy factors and changes in cardiometabolic risk factors in children.

	No Unhealthy Factor	Low Diet Quality Only	Low CRF Only	Unhealthy Sleep Pattern Only	Low CRF-Unhealthy Sleep Pattern	Low Diet Quality-Unhealthy Sleep Pattern	Low Diet Quality-Low CRF	Three Unhealthy Factors	*p*-Value *
Body composition Change in BMI									
Participants	847	308	938	744	897	226	718	571	
Mean ± SE ^†^	0.07 ± 0.03	0.12 ± 0.04	0.15 ± 0.03	0.11 ± 0.03	0.18 ± 0.03	0.18 ± 0.04	0.18 ± 0.03	0.19 ± 0.03	0.0004
Change in WC									
Participants	846	307	934	740	894	227	714	569	
Mean ± SE	0.16 ± 0.02	0.20 ± 0.03	0.20 ± 0.02	0.20 ± 0.03	0.24 ± 0.02	0.22 ± 0.04	0.21 ± 0.02	0.24 ± 0.03	0.0110
Change in PBF									
Participants	836	303	917	726	865	219	692	547	
Mean ± SE	0.17 ± 0.04	0.20 ± 0.05	0.29 ± 0.04	0.14 ± 0.04 ^c^	0.21 ± 0.04	0.24 ± 0.06	0.30 ± 0.04 ^d^	0.31 ± 0.04 ^d^	<0.0001
Blood pressure									
Change in SBP									
Participants	838	308	937	737	898	226	718	572	
Mean ± SE	−0.06 ± 0.06	0.21 ± 0.07 ^a^	−0.02 ± 0.05	−0.01 ± 0.06	−0.10 ± 0.05 ^b^	0.35 ± 0.08 ^acde^	0.22 ± 0.05 ^acde^	0.11 ± 0.06 ^ae^	<0.0001
Change in DBP									
Participants	837	308	939	739	898	227	719	573	
Mean ± SE	−0.17 ± 0.06	0.09 ± 0.07 ^a^	−0.07 ± 0.05	−0.15 ± 0.06	−0.18 ± 0.06 ^b^	0.18 ± 0.08 ^ade^	0.07 ± 0.06 ^ade^	0.05 ± 0.06 ^ae^	<0.0001
Change in MAP									
Participants	837	308	938	738	898	226	718	572	
Mean ± SE	−0.14 ± 0.06	0.15 ± 0.07 ^a^	−0.06 ± 0.05	−0.11 ± 0.06	−0.18 ± 0.06 ^b^	0.25 ± 0.08 ^acde^	0.13 ± 0.06 ^ade^	0.07 ± 0.06 ^ae^	<0.0001
Lipids									
Change in TC									
Participants	818	295	872	707	834	214	672	534	
Mean ± SE	0.07 ± 0.04	−0.01 ± 0.05	0.00 ± 0.04	0.13 ± 0.04	0.05 ± 0.04	0.02 ± 0.06	−0.13 ± 0.04 ^ade^	-0.09 ± 0.05 ^d^	<0.0001
Change in HDL-C									
Participants	819	294	872	706	834	214	673	533	
Mean ± SE	0.85 ± 0.07	0.54 ± 0.08 ^a^	0.51 ± 0.07 ^a^	0.45 ± 0.07 ^a^	0.40 ± 0.07 ^a^	0.35 ± 0.09 ^a^	0.35 ± 0.07 ^a^	0.32 ± 0.07 ^a^	<0.0001
Change in LDL-C									
Participants	820	295	874	707	832	213	673	534	
Mean ± SE	0.22 ± 0.05	-0.01 ± 0.06 ^a^	0.16 ± 0.05	0.49 ± 0.05 ^abc^	0.37 ± 0.05 ^bc^	0.18 ± 0.07 ^d^	−0.03 ± 0.05 ^acde^	0.07 ± 0.05 ^de^	<0.0001
Change in TG									
Participants	818	294	873	711	835	214	673	533	
Mean ± SE	−0.04 ± 0.06	0.08 ± 0.07	0.01 ± 0.05	0.12 ± 0.06	−0.00 ± 0.05	0.27 ± 0.07 ^a^	−0.03 ± 0.06 ^f^	0.04 ± 0.06 ^a^	0.0001
Glucose and insulin									
Change in fasting glucose									
Participants	820	295	874	711	833	214	673	533	
Mean ± SE	0.18 ± 0.06	0.20 ± 0.07	0.31 ± 0.06	0.19 ± 0.06	0.36 ± 0.06 ^ad^	0.28 ± 0.08	0.47 ± 0.06 ^abd^	0.48 ± 0.06 ^bd^	<0.0001
Change in insulin									
Participants	751	274	770	629	704	188	575	460	
Mean ± SE	−0.20 ± 0.08	0.05 ± 0.10	−0.11 ± 0.08	−0.69 ± 0.09 ^abc^	−0.51 ± 0.08 ^abc^	−0.05 ± 0.12 ^de^	−0.15 ± 0.09 ^de^	−0.15 ± 0.09 ^de^	<0.0001
Change in CMRS									
Participants	763	273	785	660	771	195	604	461	
Mean ± SE	−0.79 ± 0.15	0.04 ± 0.18 ^a^	−0.02 ± 0.15 ^a^	−0.20 ± 0.15 ^a^	0.07 ± 0.15 ^ac^	0.59 ± 0.19 ^ad^	0.41 ± 0.15 ^ad^	0.38 ± 0.16 ^ad^	<0.0001

BMI, body mass index; CMRS, cardiometabolic risk score; DBP, diastolic blood pressure; HDL-C, high-density lipoprotein cholesterol; LDL-C, low-density lipoprotein cholesterol; MAP, mean arterial pressure; SBP, systolic blood pressure; SE, standard error; TC, total cholesterol; TG, triglyceride. * General linear regression model (GLM) was used to test the difference of changes in CMR factors between individuals with a different combination of three healthy factors. We used Benjamin-Hochberg procedure to control the false discovery rate at level 5% for multiple comparisons with the *p*-value cut-off point of significance was 0.05 in the multivariable analysis. ^†^ Multivariable analysis was adjusted for age, sex, corresponding CMR factor, intervention group, puberty, grade, BMI, physical activity, and intake of energy, vegetable, fruit, pork, nuts, and legumes at baseline, birth weight, breastfeeding, household income, or parental BMI and education at baseline as fixed effects and clustering effect of children within classes in schools as random effects. Since the results are consistent between different models, this table only shows those for the multivariable analysis (Model 3). The results for Model 1 ( adjusted for age and sex at baseline as fixed effects and clustering effect of children within classes in schools as random effects) and Model 2 (adjusted for model 1 plus intervention group, puberty, grade, BMI, physical activity, and intake of energy, vegetable, fruit, pork, nuts, and legumes at baseline) were shown in Supplementary Table S4. ^abcdef^ Bonferroni post-hoc test was used to examine the difference between each two combinations of three unhealthy factors with ^a^ indicating significance compared with no unhealthy factor, ^b^ indicating significance compared with low diet quality only, ^c^ indicating significance compared with low CRF only, ^d^ indicating significance compared with unhealthy sleep pattern only, ^e^ indicating significance compared with low CRF-unhealthy sleep pattern, and ^f^ indicating significance compared with low diet quality-unhealthy sleep pattern.

## Supplementary Materials

The following are available online at https://www.mdpi.com/2072-6643/12/2/591/s1, Figure S1: Flowchart for participant selection, Table S1: P interaction for sex and unhealthy factors with changes in cardiometabolic risk factors, Table S2: P interaction for intervention and unhealthy factors with changes in cardiometabolic risk factors, Table S3: Characteristics by a combination of three unhealthy factors, Table S4: Combination of three healthy factors and changes in cardiometabolic risk factors in children, Table S5: Change in the number of unhealthy factors and changes in cardiometabolic risk factors in children, and Table S6: Combination of three healthy factors and changes in cardiometabolic risk factors in children in the control group.
