# Supplementary material for: The Clustering of Low Diet Quality, Low Physical Fitness, and Unhealthy Sleep Pattern and Its Association with Changes in Cardiometabolic Risk Factors in Children
Source: Nutrients. 2020 Feb 24;12(2):591. doi: 10.3390/nu12020591 (PMC7071380; doi:10.3390/nu12020591)
Supplement: Supplementary file 1 [file nutrients-12-00591-s001.pdf]

Supplemental Figures and Tables

Figure S1. Flowchart for participant selection

Table S1. P interaction for sex and unhealthy factors with changes in cardiometabolic risk factors

Table S2. P interaction for intervention and unhealthy factors with changes in cardiometabolic risk factors

Table S3. Characteristics by a combination of three unhealthy factors

Table S4. Combination of three healthy factors and changes in cardiometabolic risk factors in children

Table S5. Change in the number of unhealthy factors and changes in cardiometabolic risk factors in children

Table S6. Combination of three healthy factors and changes in cardiorespiratory risk factors in children in the control group

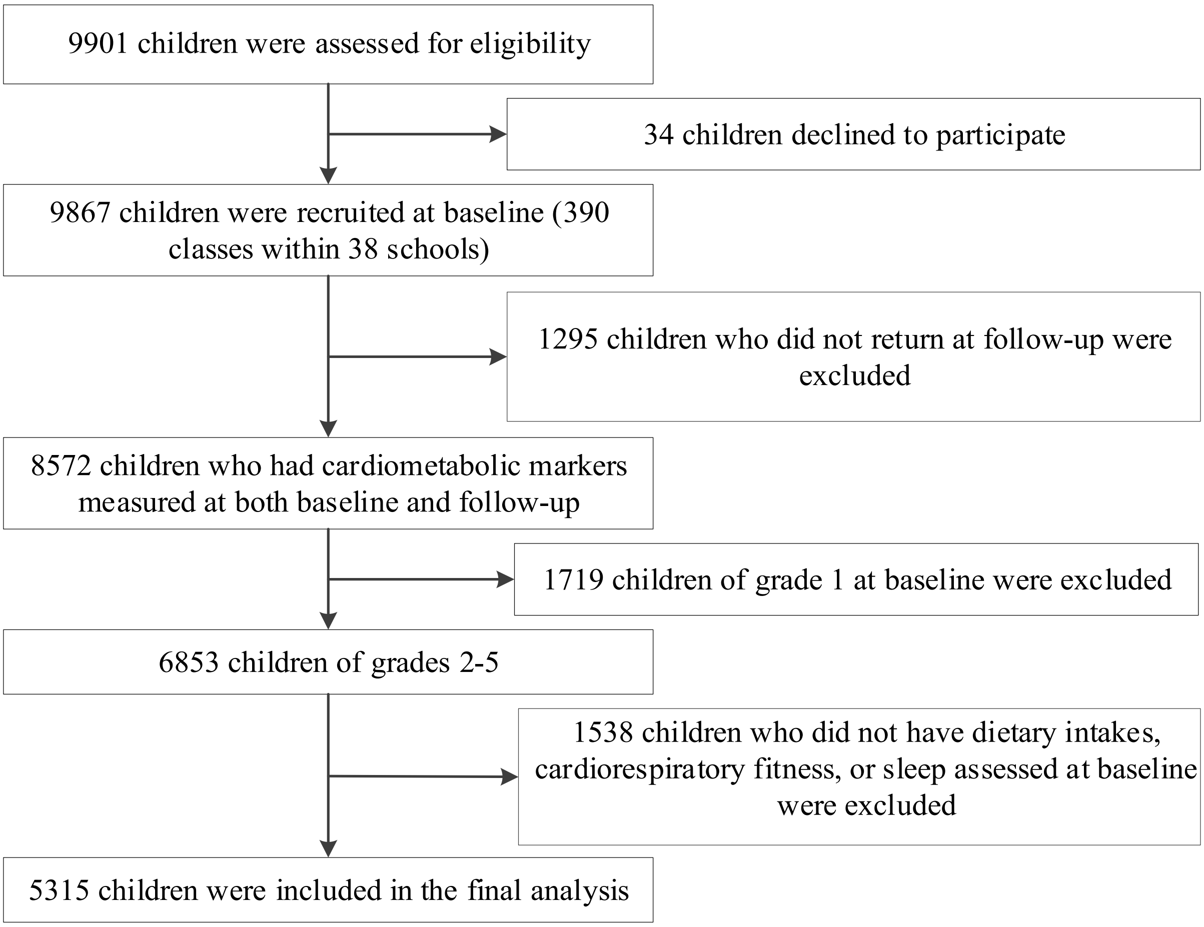
9901 children were assessed for eligibility
34 children declined to participate
9867 children were recruited at baseline (390 classes within 38 schools)
1295 children who did not return at follow-up were excluded
8572 children who had cardiometabolic markers measured at both baseline and follow-up
1719 children of grade 1 at baseline were excluded
6853 children of grades 2-5
1538 children who did not have dietary intakes, cardiorespiratory fitness, or sleep assessed at baseline were excluded
5315 children were included in the final analysis

Figure S1. Flowchart for participant selection

Table S1. P interaction for sex and unhealthy factors with changes in cardiometabolic risk factors

Changes in CMR factors	P interaction for sex and unhealthy factors-Model 1*	P interaction for sex and unhealthy factors-Model 2	P interaction for sex and unhealthy factors-Model 3†
BMI	0.0506	0.0971	0.1034
WC	0.0021	0.0222	0.0152
PBF	0.0958	0.36	0.32
SBP	0.25	0.49	0.47
DBP	0.0419	0.0468	0.0458
MAP	0.0543	0.0884	0.0833
TC	0.38	0.51	0.56
HDL-C	0.0000	0.0003	**0.0004**
LDL-C	0.14	0.17	0.18
Log TG	0.0004	0.0071	0.0130
Fasting glucose	0.66	0.78	0.81
Log insulin	0.0014	0.0092	0.0098
CMRS	0.0004	0.0016	0.0850

*GLM was used to test the interaction between sex and unhealthy factors for changes in cardiometabolic risk factors. Model 1 was adjusted for age, sex, corresponding CMR factor at baseline as fixed effect and clustering effect of children within classes in schools as random effect; Model 2 was adjusted for model 1 plus intervention group,

puberty, grade, BMI, physical activity, and intake of energy, vegetable, fruit, pork, nuts, and legumes at baseline; Model 3 was adjusted for model 2 plus birth weight, breastfeeding, household income, or parental BMI and education.

†We used Benjamin-Hochberg procedure was used to control the false discovery rate at level 5% for multiple comparisons with the P-value cut-off point of significance was 0.0038 in Model 3.

Table S2. P interaction for intervention and unhealthy factors with changes in cardiometabolic risk factors

Changes in CMR factors	P interaction for intervention and unhealthy factors Model 1*	P interaction for intervention and unhealthy factors Model 2	P interaction for intervention and unhealthy factors Model 3†
BMI	0.87	0.68	0.72
WC	0.88	0.85	0.91
PBF	0.0448	0.0217	0.0227
SBP	0.20	0.0944	0.0609
DBP	0.08	0.0714	0.0514
MAP	0.12	0.0631	0.0421
TC	0.0217	0.0134	**0.0109**
HDL-C	0.0048	0.0148	0.0185
LDL-C	0.0035	0.0023	**0.0028**
Log TG	0.36	0.61	0.68
Fasting glucose	0.0001	0.0001	**0.0001**
Insulin	0.51	0.79	0.82
CMRS	0.0706	0.0361	0.0233

*GLM was used to test the interaction between intervention and unhealthy factors for changes in cardiometabolic risk factors. Model 1 was adjusted for age, sex, corresponding CMR factor at baseline as fixed effect and clustering effect of children within classes in schools as random effect; Model 2 was adjusted for model 1 plus

intervention group, puberty, grade, BMI, physical activity, and intake of energy, vegetable, fruit, pork, nuts, and legumes at baseline; Model 3 was adjusted for model 2 plus birth weight, breastfeeding, household income, or parental BMI and education.

†We used Benjamin-Hochberg procedure was used to control the false discovery rate at level 5% for multiple comparisons with the P-value cut-off point of significance was 0.0115 in Model 3.

Table S3. Characteristics by a combination of three unhealthy factors

	No unhealthy factor	Low diet quality only	Low CRF only	Unhealthy sleep pattern only	Low PF-unhealthy sleep pattern	Low diet quality-unhealthy sleep pattern	Low diet quality-low CRF	Three unhealthy factors	P-value*
Grade									<0.0001
Two	288 (33.4)	107 (34.2)	337 (35.5)	165 (21.8)	187 (20.8)	53 (23.0)	224 (30.8)	133 (23.0)	
Three	267 (31.0)	81 (25.9)	274 (28.9)	190 (25.1)	244 (27.1)	51 (22.2)	202 (27.7)	150 (26.0)	
Four	257 (29.8)	75 (24.0)	251 (26.5)	191 (25.3)	268 (29.7)	52 (22.6)	183 (25.1)	173 (29.9)	
Five	49 (5.7)	50 (16.0)	86 (9.1)	210 (27.8)	202 (22.4)	74 (32.2)	119 (16.3)	122 (21.1)	
Birth weight†									0.0024
<2500 g	27 (3.1)	6 (1.9)	45 (4.7)	23 (3.0)	32 (3.6)	5 (2.2)	25 (3.4)	20 (3.5)	
2500-3999 g	728 (84.6)	249 (79.6)	750 (79.1)	617 (81.6)	722 (80.1)	189 (82.2)	558 (76.6)	452 (78.2)	
≥4000 g	66 (7.7)	34 (10.9)	83 (8.8)	54 (7.1)	59 (6.5)	19 (8.3)	79 (10.9)	75 (13.0)	
Missing	40 (4.6)	24 (7.7)	70 (7.4)	62 (8.2)	88 (9.8)	17 (7.4)	66 (9.1)	31 (5.4)	

Mother's BMI[‡]									<0.0001
<24 kg/m^2	717 (83.3)	235 (75.1)	726 (76.6)	619 (81.9)	655 (72.7)	181 (78.7)	551 (75.7)	417 (72.1)	
24-27.9 kg/m^2	104 (12.1)	57 (18.2)	150 (15.8)	105 (13.9)	164 (18.2)	36 (15.7)	116 (15.9)	123 (21.3)	
≥28 kg/m^2	17 (2.0)	4 (1.3)	22 (2.3)	10 (1.3)	32 (3.6)	5 (2.2)	23 (3.2)	14 (2.4)	
Missing	23 (2.7)	17 (5.4)	50 (5.3)	22 (2.9)	50 (5.5)	8 (3.5)	38 (5.2)	24 (4.2)	
Father's BMI									0.0009
<24 kg/m^2	485 (56.3)	165 (52.7)	474 (50.0)	455 (60.2)	480 (53.3)	134 (58.3)	343 (47.1)	284 (49.1)	
24-27.9 kg/m^2	292 (33.9)	108 (34.5)	339 (35.8)	230 (30.4)	294 (32.6)	75 (32.6)	271 (37.2)	215 (37.2)	
≥28 kg/m^2	61 (7.1)	23 (7.3)	85 (9.0)	49 (6.5)	77 (8.5)	13 (5.7)	76 (10.4)	55 (9.5)	
Missing	23 (2.7)	17 (5.4)	50 (5.3)	22 (2.9)	50 (5.5)	8 (3.5)	38 (5.2)	24 (4.2)	
Mother's education									0.0018
<7 years	79 (9.2)	35 (11.2)	106 (11.2)	60 (7.9)	112 (12.4)	23 (10.0)	111 (15.2)	93 (16.1)	
7-12 years	546 (63.4)	188 (60.1)	576 (60.8)	462 (61.1)	533 (59.2)	125 (54.3)	439 (60.3)	350 (60.6)	
≥13 years	207 (24.0)	69 (22.0)	198 (20.9)	189 (25.0)	187 (20.8)	68 (29.6)	118 (16.2)	100 (17.3)	
Missing	29 (3.4)	21 (6.7)	68 (7.2)	45 (6.0)	69 (7.7)	14 (6.1)	60 (8.2)	35 (6.1)	
Father's education									0.0013
<7 years	44 (5.1)	21 (6.7)	58 (6.1)	31 (4.1)	53 (5.9)	18 (7.8)	70 (9.6)	50 (8.7)	

7-12 years	560 (65.0)	190 (60.7)	586 (61.8)	461 (61.0)	572 (63.5)	142 (61.7)	454 (62.4)	388 (67.1)	
≥13 years	228 (26.5)	84 (26.8)	237 (25.0)	222 (29.4)	205 (22.8)	58 (25.2)	151 (20.7)	106 (18.3)	
Missing	29 (3.4)	18 (5.8)	67 (7.1)	42 (5.6)	71 (7.9)	12 (5.2)	53 (7.3)	34 (5.9)	
Household income per month§									0.0034
<108 USD	91 (10.6)	35 (11.2)	101 (10.7)	77 (10.2)	107 (11.9)	26 (11.3)	100 (13.7)	64 (11.1)	
109-216 USD	270 (31.4)	109 (34.8)	261 (27.5)	221 (29.2)	274 (30.4)	68 (29.6)	250 (34.3)	209 (36.2)	
217-360 USD	207 (24.0)	88 (28.1)	242 (25.5)	201 (26.6)	212 (23.5)	60 (26.1)	193 (26.5)	140 (24.2)	
≥361 USD	252 (29.3)	56 (17.9)	260 (27.4)	217 (28.7)	232 (25.7)	64 (27.8)	116 (15.9)	118 (20.4)	
Missing	41 (4.8)	25 (8.0)	84 (8.9)	40 (5.3)	76 (8.4)	12 (5.2)	69 (9.5)	47 (8.1)	
Intervention									<0.0001
No	426 (49.5)	159 (50.8)	444 (46.8)	362 (47.9)	420 (46.6)	119 (51.7)	268 (36.8)	217 (37.5)	
Yes	435 (50.5)	154 (49.2)	504 (53.2)	394 (52.1)	481 (53.4)	111 (48.3)	460 (63.2)	361 (62.5)	

BMI, body mass index; CMRS, cardiometabolic risk score; DBP, diastolic blood pressure; HDL-C, high-density lipoprotein cholesterol; LDL-C, low-density lipoprotein cholesterol; MAP, mean arterial pressure; SBP, systolic blood pressure; TC, total cholesterol; TG, triglyceride.

*Chi-square was used to test the difference of categorical variables across the healthy dietary score.

†Children were classified as low (<2500 g), normal (2500-3999 g), and high (≥4000 g) birth weight [1,2].

‡A BMI of ≦23.9 was defined as normal, 24.0 to 27.9 as overweight, and ≥28.0 as obese according to the Working Group on Obesity in China [3].

§Household income per month was divided into four groups according to the classification of total household averaged income in Urban Residents in 2007 [4].

Table S4. Combination of three healthy factors and changes in cardiometabolic risk factors in children

	No unhealthy factor	Low diet quality only	Low CRF only	Unhealthy sleep pattern only	Low CRF-unhealthy sleep pattern	Low diet quality-unhealthy sleep pattern	Low diet quality-low CRF	Three unhealthy factors	P-value*
Change in BMI									
Participants	847	308	938	744	897	226	718	571	
Mean±SE, Model 1[†]	0.01±0.02	0.07±0.04	0.09±0.02	0.06±0.02	0.13±0.02[a]	0.13±0.04	0.11±0.02	0.14±0.03	0.0002
Mean±SE, Model 2[‡]	0.02±0.02	0.08±0.03	0.11±0.02	0.06±0.02	0.14±0.02	0.13±0.04	0.14±0.02	0.16±0.03	0.0001
Change in WC									
Participants	846	307	934	740	894	227	714	569	
Mean±SE, Model 1	0.13±0.02	0.18±0.03	0.17±0.02	0.17±0.02	0.21±0.02	0.21±0.03	0.18±0.02	0.22±0.02	0.0329
Mean±SE, Model 2	0.14±0.02	0.19±0.03	0.19±0.02	0.18±0.02	0.23±0.02	0.20±0.03	0.20±0.02	0.23±0.02	0.0155
Change in PBF									
Participants	836	303	917	726	865	219	692	547	
Mean±SE, Model 1	0.18±0.03	0.22±0.05	0.30±0.03	0.13±0.03[c]	0.21±0.03	0.24±0.05	0.28±0.03	0.30±0.04[d]	<0.0001
Mean±SE, Model 2	0.17±0.03	0.20±0.05	0.28±0.03	0.12±0.03[c]	0.20±0.03	0.23±0.05	0.29±0.03[d]	0.30±0.04[d]	<0.0001

Change in SBP									
Participants	838	308	937	737	898	226	718	572	
Mean±SE, Model 1	-0.12±0.04	0.18±0.06[a]	0.00±0.04	-0.09±0.04[b]	-0.10±0.04[b]	0.30±0.07[acde]	0.24±0.04[acde]	0.16±0.05[ade]	<0.0001
Mean±SE, Model 2	-0.07±0.04	0.21±0.06[a]	-0.04±0.04	-0.02±0.04	-0.12±0.04[b]	0.34±0.07[acde]	0.21±0.04[acde]	0.10±0.05[ae]	<0.0001
Change in DBP									
Participants	837	308	939	739	898	227	719	573	
Mean±SE, Model 1	-0.22±0.04	0.08±0.06[a]	-0.06±0.04	-0.20±0.04[b]	-0.19±0.04[b]	0.14±0.07[ade]	0.10±0.04[ade]	0.09±0.05[ade]	<0.0001
Mean±SE, Model 2	-0.18±0.04	0.09±0.06[a]	-0.08±0.04	-0.15±0.05	-0.19±0.04[b]	0.17±0.07[ade]	0.07±0.05[ade]	0.05±0.05[ae]	<0.0001
Change in MAP									
Participants	837	308	938	738	898	226	718	572	
Mean±SE, Model 1	-0.19±0.04	0.13±0.06[a]	-0.04±0.04	-0.18±0.04[b]	-0.18±0.04[b]	0.21±0.07[ade]	0.16±0.04[ade]	0.11±0.05[ade]	<0.0001
Mean±SE, Model 2	-0.14±0.05	0.15±0.06[a]	-0.07±0.04	-0.12±0.05[b]	-0.18±0.04[b]	0.24±0.07[acde]	0.13±0.05[acde]	0.07±0.05[ae]	<0.0001
Change in TC									
Participants	818	295	872	707	834	214	672	534	
Mean±SE, Model 1	0.06±0.04	-0.02±0.05	-0.03±0.03	0.15±0.04[c]	0.04±0.04	0.02±0.05	-0.20±0.04[acde]	-0.15±0.04[de]	<0.0001
Mean±SE, Model 2	0.05±0.03	-0.04±0.05	-0.02±0.03	0.11±0.04	0.03±0.03	0.00±0.05	-0.17±0.04[ade]	-0.13±0.04[d]	<0.0001
Change in HDL-C									
Participants	819	294	872	706	834	214	673	533	

Mean±SE, Model 1	1.02±0.06	0.63±0.07[a]	0.59±0.05[a]	0.60±0.06[a]	0.48±0.06[a]	0.43±0.08[a]	0.37±0.06[acd]	0.33±0.06[abcd]	<0.0001
Mean±SE, Model 2	0.91±0.06	0.58±0.07[a]	0.56±0.06[a]	0.49±0.06[a]	0.44±0.06[a]	0.41±0.08[a]	0.38±0.06[a]	0.37±0.06[a]	<0.0001
Change in LDL-C									
Participants	820	295	874	707	832	213	673	534	
Mean±SE, Model 1	0.22±0.04	-0.05±0.06[a]	0.14±0.04	0.53±0.04[abc]	0.40±0.04[abc]	0.19±0.06[d]	-0.07±0.04[acdef]	0.04±0.05[de]	<0.0001
Mean±SE, Model 2	0.26±0.04	-0.00±0.05[a]	0.20±0.04	0.53±0.04[abc]	0.41±0.04[bc]	0.21±0.06[d]	-0.01±0.04[acde]	0.08±0.04[de]	<0.0001
Change in TG									
Participants	818	294	873	711	835	214	673	533	
Mean±SE, Model 1	-0.11±0.05	0.05±0.06	-0.00±0.04	0.05±0.05	-0.00±0.04	0.25±0.07[a]	-0.05±0.05[f]	0.06±0.05[a]	<0.0001
Mean±SE, Model 2	-0.03±0.04	0.08±0.06	0.01±0.04	0.13±0.05	0.00±0.04	0.26±0.07[a]	-0.03±0.05[f]	0.03±0.05[a]	0.0001
Change in fasting glucose									
Participants	820	295	874	711	833	214	673	533	
Mean±SE, Model 1	0.17±0.05	0.18±0.07	0.31±0.05	0.19±0.06	0.35±0.05[ad]	0.25±0.07	0.47±0.06[abcd]	0.46±0.06[bd]	<0.0001
Mean±SE, Model 2	0.19±0.06	0.22±0.07	0.33±0.05	0.21±0.06	0.37±0.06[ad]	0.29±0.07	0.48±0.06[abd]	0.49±0.06[bd]	<0.0001
Change in insulin									
Participants	751	274	770	629	704	188	575	460	

Mean±SE, Model 1	-0.23±0.07	0.08±0.09	-0.02±0.06	-0.81±0.07[abc]	-0.51±0.07[bc]	-0.08±0.11[d]	-0.06±0.07[de]	-0.03±0.08[de]	<0.0001
Mean±SE, Model 2	-0.17±0.06	0.09±0.09	-0.09±0.06	-0.67±0.07[abc]	-0.49±0.06[abc]	-0.05±0.10[d]	-0.12±0.07[de]	-0.12±0.07[de]	<0.0001
Change in CMRS									
Participants	763	273	785	660	771	195	604	461	
Mean±SE, Model 1	-1.16±0.13	-0.16±0.17[a]	-0.13±0.13[a]	-0.52±0.13[a]	-0.03±0.13[acd]	0.37±0.19[ad]	0.37±0.13[acd]	0.42±0.14[acd]	<0.0001
Mean±SE, Model 2	-0.86±0.12	-0.01±0.16[a]	-0.10±0.12[a]	-0.27±0.13[a]	0.00±0.12[ac]	0.49±0.18[ad]	0.37±0.13[ad]	0.31±0.13[ad]	<0.0001

BMI, body mass index; CMRS, cardiometabolic risk score; DBP, diastolic blood pressure; HOMA-IR, homeostatic model assessment of insulin resistance; HDL-C, high-density lipoprotein cholesterol; LDL-C, low-density lipoprotein cholesterol; MAP, mean arterial pressure; SBP, systolic blood pressure; SE, standard error; TC, total cholesterol; TG, triglyceride.

* General linear regression model (GLM) was used to test the difference of changes in CMR factors between individuals with a different combination of three healthy factors. We used the Benjamin-Hochberg procedure was used to control the false discovery rate at level 5% for multiple comparisons with the P-value cut-off point of significance was 0.05 in Model 3.

[†] Model 1 was adjusted for age, sex, corresponding CMR factor at baseline as fixed effects and clustering effect of children within classes in schools as random effects.

[‡] Model 2 was adjusted for model 1 plus intervention group, puberty, grade, BMI, physical activity, and intake of energy, vegetable, fruit, pork, nuts and legumes at baseline.

[abcdef] Bonferroni Post-hoc test was used to examine the difference between each two combinations of three unhealthy factors with [a] indicating significance compared with no unhealthy factor, [b] indicating significance compared with low diet quality only, [c] indicating significance compared with low CRF only, [d] indicating significance compared with unhealthy sleep pattern only, [e] indicating significance compared with low CRF-unhealthy sleep pattern, and [f] indicating significance compared with low diet quality-unhealthy sleep pattern.

Table S5. Change in the number of unhealthy factors and changes in cardiometabolic risk factors in children

	Change in the number of unhealthy factors			
	Increase	No change	Increase	P-trend
Change in BMI				
Participants	703	1157	604	
Mean ± SE, Model 1[†]	0.12 ± 0.03	0.09 ± 0.02	-0.01 ± 0.03[ab]	0.0002
Mean ± SE, Model 2[‡]	0.14 ± 0.03	0.10 ± 0.02	-0.00 ± 0.03[ab]	0.0001
Mean ± SE, Model 3[§]	0.15 ± 0.04	0.11 ± 0.04	0.00 ± 0.04[ab]	<0.0001
Change in WC				
Participants	701	1149	601	
Mean ± SE, Model 1	0.22 ± 0.02	0.19 ± 0.02	0.11 ± 0.03[ab]	0.0007
Mean ± SE, Model 2	0.23 ± 0.02	0.20 ± 0.02	0.12 ± 0.02[ab]	0.0010
Mean ± SE, Model 3	0.24 ± 0.03	0.21 ± 0.03	0.13 ± 0.03[ab]	0.0010
Change in PBF				
Participants	689	1120	587	
Mean ± SE, Model 1	0.19 ± 0.04	0.18 ± 0.03	0.16 ± 0.04	0.58
Mean ± SE, Model 2	0.17 ± 0.04	0.15 ± 0.04	0.12 ± 0.04	0.34
Mean ± SE, Model 3	0.17 ± 0.06	0.14 ± 0.05	0.12 ± 0.06	0.32
Change in SBP				
Participants	699	1153	601	
Mean ± SE, Model 1	-0.06 ± 0.05	-0.06 ± 0.04	-0.12 ± 0.05	0.43
Mean ± SE, Model 2	-0.08 ± 0.06	-0.05 ± 0.05	-0.10 ± 0.06	0.77
Mean ± SE, Model 3	-0.09 ± 0.08	-0.05 ± 0.07	-0.11 ± 0.08	0.83
Change in DBP				

Participants	698	1155	602	
Mean ± SE, Model 1	-0.17 ± 0.06	-0.20 ± 0.05	-0.20 ± 0.06	0.71
Mean ± SE, Model 2	-0.16 ± 0.06	-0.18 ± 0.05	-0.18 ± 0.06	0.72
Mean ± SE, Model 3	-0.20 ± 0.08	-0.22 ± 0.08	-0.23 ± 0.08	0.68
Change in MAP				
Participants	698	1154	601	
Mean ± SE, Model 1	-0.15 ± 0.06	-0.16 ± 0.05	-0.18 ± 0.06	0.61
Mean ± SE, Model 2	-0.14 ± 0.06	-0.14 ± 0.05	-0.17 ± 0.06	0.74
Mean ± SE, Model 3	-0.18 ± 0.08	-0.17 ± 0.08	-0.20 ± 0.08	0.73
Change in TC				
Participants	656	1113	568	
Mean ± SE, Model 1	0.08 ± 0.04	0.02 ± 0.03	-0.02 ± 0.04	0.0381
Mean ± SE, Model 2	0.08 ± 0.04	0.03 ± 0.03	-0.02 ± 0.04	0.0405
Mean ± SE, Model 3	0.08 ± 0.05	0.03 ± 0.05	-0.01 ± 0.05	0.0645
Change in HDL-C				
Participants	657	1113	566	
Mean ± SE, Model 1	0.53 ± 0.06	0.67 ± 0.05[a]	0.95 ± 0.07[ab]	<0.0001
Mean ± SE, Model 2	0.52 ± 0.06	0.62 ± 0.05	0.84 ± 0.07[ab]	<0.0001
Mean ± SE, Model 3	0.40 ± 0.08	0.49 ± 0.08	0.72 ± 0.09[ab]	<0.0001
Change in LDL-C				
Participants	657	1115	568	
Mean ± SE, Model 1	0.32 ± 0.06	0.31 ± 0.06	0.19 ± 0.06[b]	0.0251
Mean ± SE, Model 2	0.39 ± 0.06	0.38 ± 0.05	0.25 ± 0.06[b]	0.0129
Mean ± SE, Model 3	0.31 ± 0.07	0.31 ± 0.07	0.18 ± 0.07[b]	0.0269
Change in TG				

Participants	656	1117	569	
Mean ± SE, Model 1	0.18 ± 0.04	0.10 ± 0.04	-0.08 ± 0.04[ab]	<0.0001
Mean ± SE, Model 2	0.19 ± 0.04	0.13 ± 0.04	-0.03 ± 0.05[ab]	0.0002
Mean ± SE, Model 3	0.14 ± 0.06	0.09 ± 0.06	-0.08 ± 0.07[ab]	0.0002
Change in fasting glucose				
Participants	659	1117	568	
Mean ± SE, Model 1	-0.03 ± 0.06	0.09 ± 0.06[a]	0.03 ± 0.06	0.09
Mean ± SE, Model 2	-0.02 ± 0.06	0.09 ± 0.06[a]	0.02 ± 0.07	0.28
Mean ± SE, Model 3	-0.08 ± 0.07	0.03 ± 0.07[a]	-0.03 ± 0.08	0.25
Change in insulin				
Participants	587	1015	517	
Mean ± SE, Model 1	-0.60 ± 0.09	-0.64 ± 0.08	-0.37 ± 0.10[b]	0.0551
Mean ± SE, Model 2	-0.60 ± 0.09	-0.60 ± 0.08	-0.34 ± 0.10[b]	0.0236
Mean ± SE, Model 3	-0.57 ± 0.12	-0.58 ± 0.12	-0.31 ± 0.13[b]	0.0224
Change in CMRS				
Participants	612	1024	523	
Mean ± SE, Model 1	-0.39 ± 0.14	-0.52 ± 0.12	-1.06 ± 0.14[ab]	<0.0001
Mean ± SE, Model 2	-0.39 ± 0.14	-0.43 ± 0.13	-0.93 ± 0.15[ab]	0.0005
Mean ± SE, Model 3	-0.35 ± 0.18	-0.39 ± 0.17	-0.89 ± 0.19[ab]	0.0005

BMI, body mass index; CMRS, cardiometabolic risk score; DBP, diastolic blood pressure; HOMA-IR, homeostatic model assessment of insulin resistance; HDL-C, high-density lipoprotein cholesterol; LDL-C, low-density lipoprotein cholesterol; MAP, mean arterial pressure; SBP, systolic blood pressure; SE, standard error; TC, total cholesterol; TG, triglyceride.

* General linear regression model (GLM) was used to test the difference of changes in CMR factors between individuals in different groups of change in the number of unhealthy factors. We used the Benjamin-Hochberg procedure was used to control the false discovery rate at level 5% for multiple comparisons with the P-value cut-off point of significance was 0.0231 in Model 3.

† Model 1 was adjusted for age, sex, corresponding CMR factor at baseline as fixed effects and clustering effect of children within classes in schools as random effects.

‡ Model 2 was adjusted for model 1 plus intervention group, puberty, grade, BMI, physical activity, and intake of energy, vegetable, fruit, pork, nuts and legumes at baseline.

§ Model 3 was adjusted for model 2 plus birth weight, breastfeeding, household income, or parental BMI and education.

[ab] Bonferroni Post-hoc test was used to examine the difference between every two groups with [a] indicating significance compared with the increase in the number of unhealthy factors, and [b] indicating significance compared with no change in the number of unhealthy factors.

Table S6. Combination of three healthy factors and changes in cardiometabolic risk factors in children in the control group

	No unhealthy factor	Low diet quality only	Low CRF only	Unhealthy sleep pattern only	Low CRF-unhealthy sleep pattern	Low diet quality-unhealthy sleep pattern	Low diet quality-low CRF	Three unhealthy factors	P-value*
Change in BMI									
Participants	420	156	440	358	417	117	264	214	
Mean ± SE, Model 1[†]	0.01±0.03	0.10±0.05	0.09±0.03	0.10±0.03	0.13±0.03	0.18±0.06	0.12±0.04	0.16±0.04	0.0514
Mean ± SE, Model 2[‡]	0.04±0.03	0.12±0.05	0.11±0.03	0.12±0.03	0.16±0.03	0.19±0.05	0.15±0.04	0.17±0.04	0.0946
Mean ± SE, Model 3[§]	0.08±0.05	0.14±0.06	0.15±0.04	0.16±0.05	0.19±0.04	0.23±0.06	0.18±0.05	0.19±0.05	0.19
Change in WC									
Participants	421	156	439	357	416	119	262	214	
Mean ± SE, Model 1	0.14±0.03	0.20±0.04	0.19±0.03	0.20±0.03	0.24±0.03	0.23±0.05	0.23±0.03	0.28±0.04	0.0596
Mean ± SE, Model 2	0.17±0.02	0.22±0.04	0.22±0.02	0.23±0.03	0.27±0.02	0.25±0.04	0.24±0.03	0.29±0.03	0.0439
Mean ± SE, Model 3	0.19±0.04	0.23±0.05	0.23±0.03	0.24±0.04	0.29±0.03	0.27±0.05	0.26±0.04	0.31±0.04	0.0761
Change in PBF									
Participants	413	153	433	348	406	117	254	202	
Mean ± SE, Model 1	0.25±0.04	0.31±0.06	0.27±0.04	0.11±0.04	0.19±0.04	0.31±0.07	0.31±0.05	0.37±0.05[d]	0.0008
Mean ± SE, Model 2	0.25±0.04	0.30±0.06	0.23±0.04	0.14±0.04	0.19±0.04	0.31±0.07	0.29±0.05	0.34±0.05	0.0199

Mean ± SE, Model 3	0.24±0.06	0.27±0.07	0.23±0.05	0.13±0.06	0.17±0.05	0.30±0.08	0.28±0.06	0.32±0.06	0.0315
Change in SBP									
Participants	417	157	439	357	418	119	263	216	
Mean ± SE, Model 1	-0.03±0.06	0.21±0.09	0.03±0.06	-0.01±0.07	-0.09±0.06	0.21±0.10	0.21±0.07	0.18±0.08	0.0008
Mean ± SE, Model 2	0.06±0.06	0.27±0.09	-0.01±0.06	0.07±0.07	-0.12±0.06[b]	0.29±0.10	0.16±0.07	0.10±0.08	0.0002
Mean ± SE, Model 3	-0.07±0.08	0.13±0.10	-0.13±0.08	-0.06±0.09	-0.24±0.08	0.15±0.11	0.03±0.09	-0.04±0.09	0.0006
Change in DBP									
Participants	416	157	440	358	418	119	263	216	
Mean ± SE, Model 1	-0.10±0.06	0.17±0.09	0.02±0.06	-0.06±0.06	-0.14±0.06	0.13±0.10	0.04±0.07	0.15±0.08	0.0016
Mean ± SE, Model 2	-0.04±0.06	0.21±0.09	-0.01±0.06	-0.00±0.07	-0.17±0.06	0.20±0.10	0.02±0.07	0.11±0.08	0.0012
Mean ± SE, Model 3	-0.16±0.08	0.08±0.10	-0.12±0.08	-0.13±0.08	-0.28±0.08	0.08±0.11	-0.11±0.09	-0.02±0.09	0.0028
Change in MAP									
Participants	416	157	439	357	418	119	263	216	
Mean ± SE, Model 1	-0.07±0.06	0.21±0.09	0.02±0.06	-0.05±0.07	-0.14±0.06	0.18±0.10	0.11±0.07	0.17±0.08	0.0004
Mean ± SE, Model 2	0.00±0.06	0.26±0.09	-0.01±0.06	0.02±0.07	-0.17±0.06[b]	0.26±0.10[e]	0.07±0.07	0.11±0.08	0.0002
Mean ± SE, Model 3	-0.13±0.08	0.11±0.10	-0.14±0.08	-0.12±0.09	-0.30±0.08[b]	0.12±0.11	-0.07±0.09	-0.04±0.09	0.0004
Change in TC									
Participants	403	147	416	335	389	111	247	194	
Mean ± SE, Model 1	0.13±0.06	0.08±0.08	0.09±0.06	0.13±0.06	0.13±0.06	0.07±0.08	0.03±0.07	-0.01±0.07	0.32

Mean ± SE, Model 2	0.14±0.06	0.10±0.08	0.12±0.06	0.10±0.06	0.13±0.06	0.07±0.08	0.05±0.07	0.00±0.07	0.45
Mean ± SE, Model 3	0.09±0.07	0.05±0.08	0.07±0.07	0.05±0.07	0.08±0.07	0.02±0.09	-0.01±0.08	-0.04±0.08	0.51
Change in HDL-C									
Participants	405	147	415	337	390	111	248	193	
Mean ± SE, Model 1	0.82±0.08	0.65±0.10	0.59±0.08	0.61±0.08	0.57±0.08	0.59±0.11	0.38±0.09^{a}	0.31±0.09	<0.0001
Mean ± SE, Model 2	0.75±0.08	0.61±0.10	0.58±0.08	0.51±0.08	0.54±0.08	0.54±0.11	0.40±0.09^{a}	0.35±0.09	0.0005
Mean ± SE, Model 3	0.71±0.10	0.58±0.11	0.55±0.09	0.49±0.10	0.52±0.10	0.50±0.12	0.38±0.10	0.33±0.11	0.0009
Change in LDL-C									
Participants	405	147	416	335	388	111	248	194	
Mean ± SE, Model 1	0.12±0.06	0.00±0.07	0.10±0.05	0.39±0.06^{abc}	0.32±0.05^{b}	0.20±0.08	0.07±0.06^{de}	0.09±0.07^{d}	<0.0001
Mean ± SE, Model 2	0.18±0.05	0.07±0.07	0.18±0.05	0.40±0.06^{b}	0.35±0.05	0.25±0.08	0.12±0.06^{d}	0.13±0.06	<0.0001
Mean ± SE, Model 3	0.11±0.07	0.01±0.08	0.11±0.06	0.32±0.07^{b}	0.28±0.07	0.17±0.09	0.06±0.07	0.07±0.07	<0.0001
Change in TG									
Participants	405	146	417	337	390	111	248	193	
Mean ± SE, Model 1	-0.07±0.07	0.01±0.09	0.08±0.07	0.04±0.07	0.02±0.07	0.13±0.10	0.13±0.08	0.24±0.08	0.0119

Mean ± SE, Model 2	0.01±0.07	0.05±0.09	0.07±0.07	0.13±0.07	0.01±0.07	0.18±0.10	0.09±0.07	0.16±0.08	0.28
Mean ± SE, Model 3	-0.04±0.08	0.00±0.10	0.02±0.08	0.07±0.09	-0.05±0.08	0.12±0.11	0.02±0.09	0.10±0.09	0.35
Change in fasting glucose									
Participants	405	147	416	337	389	111	248	194	
Mean ± SE, Model 1	0.35±0.08	0.30±0.09	0.41±0.08	0.45±0.08	0.43±0.08	0.42±0.10	0.35±0.09	0.45±0.09	0.26
Mean ± SE, Model 2	0.35±0.08	0.31±0.09	0.43±0.08	0.43±0.08	0.44±0.08	0.41±0.10	0.37±0.09	0.48±0.09	0.30
Mean ± SE, Model 3	0.35±0.09	0.32±0.10	0.43±0.09	0.43±0.09	0.44±0.09	0.42±0.11	0.36±0.10	0.49±0.10	0.30
Change in insulin									
Participants	358	136	374	300	342	94	220	176	
Mean ± SE, Model 1	-0.33±0.10	-0.03±0.14	-0.06±0.09	-0.92±0.10[abc]	-0.54±0.10	-0.18±0.16[d]	0.03±0.11[de]	0.05±0.12[de]	<0.0001
Mean ± SE, Model 2	-0.28±0.10	0.03±0.13	-0.17±0.09	-0.77±0.10[abc]	-0.56±0.09[b]	-0.10±0.15[d]	-0.05±0.11[de]	-0.08±0.12[d]	<0.0001
Mean ± SE, Model 3	-0.29±0.13	0.01±0.15	-0.18±0.12	-0.78±0.13[abc]	-0.57±0.12	-0.09±0.17[d]	-0.08±0.13[d]	-0.10±0.14[d]	<0.0001
Change in CMRS									
Participants	379	135	372	317	359	106	214	161	

Mean ± SE, Model 1	-0.59±0.20	0.06±0.25	0.10±0.19[a]	-0.08±0.20	0.02±0.20	0.33±0.26	0.41±0.22[a]	0.61±0.23	<0.000<br>1
Mean ± SE, Model 2	-0.34±0.19	0.22±0.23	0.12±0.18	0.16±0.19	0.02±0.19	0.48±0.25	0.34±0.21	0.46±0.22	0.0011
Mean ± SE, Model 3	-0.37±0.23	0.19±0.26	0.09±0.22	0.11±0.23	-0.03±0.22	0.47±0.28	0.28±0.24	0.44±0.25	0.0013

BMI, body mass index; CMRS, cardiometabolic risk score; DBP, diastolic blood pressure; HOMA-IR, homeostatic model assessment of insulin resistance; HDL-C, high-density lipoprotein cholesterol; LDL-C, low-density lipoprotein cholesterol; MAP, mean arterial pressure; SBP, systolic blood pressure; SE, standard error; TC, total cholesterol; TG, triglyceride.

* General linear regression model (GLM) was used to test the difference of changes in CMR factors between individuals with a different combination of three healthy factors. We used the Benjamin-Hochberg procedure was used to control the false discovery rate at level 5% for multiple comparisons with the P-value cut-off point of significance was 0.0307 in Model 3.

† Model 1 was adjusted for age, sex, corresponding CMR factor at baseline as fixed effects and clustering effect of children within classes in schools as random effects.

‡ Model 2 was adjusted for model 1 plus intervention group, puberty, grade, BMI, physical activity, and intake of energy, vegetable, fruit, pork, nuts and legumes at baseline.

§ Model 3 was adjusted for model 2 plus birth weight, breastfeeding, household income, or parental BMI and education.

[abcde] Bonferroni Post-hoc test was used to examine the difference between each two combinations of three unhealthy factors with [a] indicating significance compared with no unhealthy factor, [b] indicating significance compared with low diet quality only, [c] indicating significance compared with low CRF only, [d] indicating significance compared with unhealthy

sleep pattern only, and [e] indicating significance compared with low CRF-unhealthy sleep pattern.

References

1. Hughes, M.M.; Black, R.E.; Katz, J. 2500-g Low Birth Weight Cutoff: History and Implications for Future Research and Policy. *Maternal and child health journal* **2017**, *21*, 283-289, doi:10.1007/s10995-016-2131-9.
2. Choukem, S.P.; Njim, T.; Atashili, J.; Hamilton-Shield, J.P.; Mbu, R. High birth weight in a suburban hospital in Cameroon: an analysis of the clinical cut-off, prevalence, predictors and adverse outcomes. *BMJ open* **2016**, *6*, e011517, doi:10.1136/bmjopen-2016-011517.
3. Chen, C.; Lu, F.C. The guidelines for prevention and control of overweight and obesity in Chinese adults. *Biomed. Environ. Sci.* **2004**, *17 Suppl*, 1-36.
4. China, N.B.o.S.o. Classification of total househol averaged income in Urban Residents in 2007. National Bureau of Statistics of China: Beijing, 2007; http://data.stats.gov.cn/easyquery.htm?cn=C01.
